# Notes on the genus Ismarus Haliday (Hymenoptera, Diapriidae) from China

**DOI:** 10.3897/zookeys.108.768

**Published:** 2011-06-17

**Authors:** Jing-xian Liu, Hua-yan Chen, Zai-fu Xu

**Affiliations:** Department of Entomology, College of Natural Resources and Environment, South China Agricultural University, Guangzhou 510640, the People’s Republic of China

**Keywords:** Ismarinae, parasitoids, new species, Oriental

## Abstract

The Chinese species of the genus *Ismarus* Haliday, 1835, are revised for the first time. Three new species from the Oriental region of China and belonging to *Ismarus halidayi*-group are described and illustrated: *Ismarus longus* **sp. n.**, *Ismarus nigritrochanter* **sp. n.** and *Ismarus parvicellus* **sp. n.** Two species are newly reported for the Chinese fauna: *Ismarus dorsiger* (Haliday, 1831) and *Ismarus halidayi* Foerster, 1850. A key to the Chinese species of the genus is provided. The type specimens are deposited in the Hymenopteran Collection of South China Agricultural University, Guangzhou (SCAU).

## Introduction

The small subfamily Ismarinae belongs to the family of Diapriidae and is characterized by the rather low insertion of antennae, the transverse head, the reduced notauli, the fore legs with a peculiar combing apparatus, the hind tibia with a false second spur and the carapace-like metasoma. The subfamily includes two genera: *Ismarus* Haliday, 1835 and *Szelenyioprioides* Szabó, 1974. The relationships between Ismarinae and the other subfamilies of the family Diapriidae have been discussed by Masner (1976). In total, the Ismarinae contains thirty species (twenty nine in *Ismarus* and one in *Szelenyioprioides*) described from the Palaearctic, Nearctic, Neotropical and Australian regions (Nixon 1957; Hellén 1964; Wall 1967a, b; Szabó 1974; Masner 1976; Johnson 1992; Ventura et al. 1997; Notton 2007). Formally no species are known from the Oriental and Ethiopian regions, but Masner (1976) mentioned some undescribed species from both regions.

The subfamily Ismarinae has been studied mainly in the Holarctic and Neotropical regions, with no species known from China prior to this study. During recent years, we have accumulated many specimens of Diapriidae during our survey of the Hymenoptera of China. Among them, twenty five specimens belonging to the genus *Ismarus* represent five species, of which three species from the Oriental region of China are described as new to science in this paper and two species are newly reported from the Oriental and Palaearctic regions of China.

## Materials and methods

Examined specimens were collected by sweeping and by yellow pan traps (YPT) from different provinces of China between 2006 and 2010. All specimens are deposited in the Hymenopteran Collection of South China Agricultural University, Guangzhou. For the examination, an Olympus stereomicroscope was used. The photographs are made by a digital camera (Q-Imaging, RTV) mounted on a Zeiss stereomicroscope and with Image-Pro Plus software.

For the used terminology sees Masner (1976). Abbreviations used in the text are: POL= postocellar line or shortest distance between both posterior ocelli; OOL= oculo-ocellar line or shortest distance between posterior ocellus and corresponding compound eye.

## Results

### Genus Ismarus

Haliday, 1835

http://species-id.net/wiki/Ismarus

Ismarus Haliday 1835, Entomological Magazine 2: 467. Type: *Cinetus dorsiger* Haliday, by monotypy.
Entomia Herrich-Schäffer 1840, Nomenclator Entomologicus 2: 127. Type: *Entomia campanulata* Herrich-Schäffer, 1840, by monotypy.
Entomius Haliday 1857, Nature History Review 4: 169. Emendation.
Agonophorus Dahlbom 1858, Öfversigt af Kongliga Ventenskaps-Akadamiens Förhandlingar 14: 289. Synonymized by Dessart (1967). Type: *Ismarus rugulosus* Foerster, designated by Muesebeck (1972).
Ismarus Haliday, 1835: Masner 1976, Canadian Entomologist 108: 1251.
Ismarus Haliday, 1835: Johnson 1992, Memoirs of the American Entomological Institute 51: 259.

#### Description.

Body stout; body colour usually black, but brown in a few species. Head transverse in dorsal view, with sparse setae on face, frons and occiput; labrum exposed and sclerotized; mandible bidentate; palpal formula 4-3 or 5-3; antennae inserted low on face, close to clypeus; face with a distinct transverse carina below antennal sockets; antennal shelf not prominent; antenna of female 15-segmented, of male 14-segmented; modified male sex-segment is second flagellomere, rarely both first and second flagellomeres modified; eyes bare; occipital carina complete; pronotum dorsally and along anterior margin with long setae; mesoscutum convex, smooth; notauli reduced to anterior pits; humeral sulcus developed; anterior scutellar pit with a weak but distinct median longitudinal carina; scutellum posteriorly raised, with posterior margin truncate or round; metanotum crenulated, with median carina distinct; propodeum with distinct transverse and longitudinal carinae; metapleuron reticulate rugose, with dense setosity; fore wing with radial cell closed; fore tibia with regular spur and one false spur, hind tibia strongly incrassate; petiole short and transverse; base of second tergite with longitudinal furrows; sutures between tergites distinct or absent; sternite with fine setae.

#### Biology.

Some species of the genus are hyperparasitoids of Dryinidae (Hymenoptera). *Ismarus flavicornis* (Thomson, 1859) was reared from *Anteon flavicorne* (Dalman, 1818), *Ismarus halidayi* was reared from an *Anteon* sp. and *Ismarus dorsiger* was recorded to attack an *Aphelopus* sp. (Chambers 1955; 1981; Nixon 1957; Jervis 1979).

#### Distribution.

Palaearctic, Oriental, Ethiopian, Nearctic, Neotropical and Australian regions.

#### Discussion.

Masner (1976) divided *Ismarus* into four species groups: *Ismarus rugulosus*-, *Ismarus halidayi*-, *Ismarus rex*- and *Ismarus dorsiger*-groups. The species of the present study belong to the *Ismarus halidayi*- and *Ismarus dorsiger*-groups. Compared to the diagnosis of *Ismarus* given by Masner (1976), we found that all species from China have the labrum exposed and possess a transverse carina on the lower face just below the antennal sockets.

### Ismarus longus

Liu, Chen & Xu sp. n.

urn:lsid:zoobank.org:act:B3D908D7-C636-4EAE-BE02-A6D2CA719FF2

http://species-id.net/wiki/Ismarus_longus

Figs 1–4 

#### Holotype.

Female. Body length 3.2 mm; fore wing length 3.0 mm.

#### Description.

*Head*. Head in dorsal view 2.0 times as wide as long; vertex abruptly sloping behind ocelli; temple narrowed behind eyes; occipital carina strong and complete, not crenulate; POL equals to OOL; toruli separated from each other; face setose; clypeus evenly convex; epistomal sulcus distinct; eyes bare, eye height 4.5 times length of malar space; malar sulcus absent; frons setose just above antennal sockets. Antenna 1.1 times as long as body; scape cylindrical, with apical rim simple; pedicel basally attenuate and apically broad; ratios of length to width of antennal segments: 20 : 6; 9 : 5; 16 : 4; 20 : 4; 13 : 4; 13 : 4; 10 : 5; 10 : 5; 9 : 5; 9 : 5; 9 : 5; 9 : 5; 9 : 5; 9 : 5; 13 : 5.

*Mesosoma*. Mesosoma in dorsal view 0.8 times as long as width of head; pronotum angular in dorsal view, anteriorly rugose-punctate and setose; central part of lateral side of pronotum smooth, with anterior and upper margins rugose-punctate and setose; mesoscutum smooth and convex, with some sparse long setae near humeral sulcus and notauli; notauli anteriorly present, oblique long and pit-like, crenulate inside; humeral sulcus deep and crenulate, 1.4 times length of tegula; anterior scutellar pit crenulate, with weak median longitudinal carina; scutellum smooth, posterior rim rounded; propodeum rugose, with transverse and longitudinal carinae present; mesopleuron smooth and bare, with upper corner below tegula punctate and setose; metapleuron reticulate-rugose and setose.

*Wings*. Fore wing with costal, subcostal, basal, marginal, stigmal and postmarginal veins tubular; radial cell closed, 0.6 times length of marginal vein and 3.0 times as long as its height. Hind wing with a basal cell.

*Legs*. Fore and middle legs slender; hind tibia strongly incrassate.

*Metasoma*. Petiole transverse, weakly rugose and with longitudinal carinae; second tergite smooth and scattered with a few setae along lower side, median furrow extending to 0.4 length of second tergite; sutures between tergites complete and well impressed; sternites finely punctate and setose.

#### Colour.

Body black. Antenna black, with scape brown, pedicel and first flagellomere dark brown; mandible black with its apical half reddish brown; palpi black; tegula blackish brown. Legs brown, all coxae black but apically brown; hind femur brown with incrassate part black; hind tibia black with basal 0.2 reddish brown; hind tarsus reddish brown with first tarsomere black. Wings hyaline, veins blackish brown.

#### Male.

Unknown.

#### Distribution.

China (Yunnan).

#### Material examined.

Holotype, female, CHINA: Yunnan, Yingjiang, Tongbiguan (24.60°N, 97.65°E), 2009.V.20, Man-man Wang, No. 200900933. Paratypes: 1 female, same data as type, No. 200900492; 1 female, Yunnan, Tengchong, Jietou Town (25.40°N, 98.70°E), 2009.V.13, Man-man Wang, No. 200902486; 1 female, Yunnan, Tengchong, Jietou Town (25.40°N, 98.70°E), 2009.V.12, Jie Zeng, No. 200902519.

#### Diagnosis.

This species belongs to the *Ismarus halidayi*-group of Manser (1976) and is similar to *Ismarus halidayi* Foerster, 1850, but it differs from the latter by having the second flagellomere 5.0 times as long as wide (2.6 times as long as wide in *Ismarus halidayi*), apical antennal segment 2.6 times as long as wide (2.0 times); notauli anteriorly present, oblique long and pit-like, crenulate inside (smooth) and the radial cell of fore wing shorter than marginal vein (radial cell as long as marginal vein).

#### Etymology.

The specific name refers to the long second flagellomere.

**Figures 1–4.**
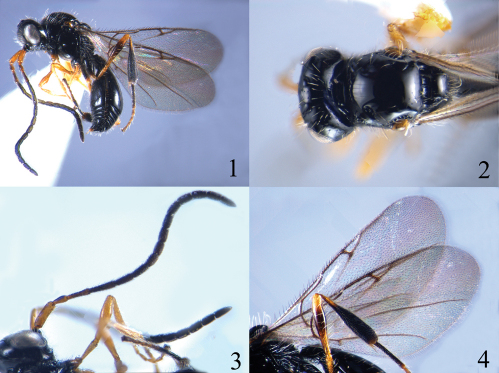
*Ismarus longus* sp. n. **1** habitus, lateral view **2** head and thorax, dorsal view **3** antennae **4** fore wings and hind leg.

### Ismarus nigritrochanter

Liu, Chen & Xu sp. n.

urn:lsid:zoobank.org:act:4F711F1A-7878-49CB-B18B-6977B824612F

http://species-id.net/wiki/Ismarus_nigritrochanter

Figs 5–8 

#### Holotype.

Female. Body length 3.1 mm; fore wing length 3.1 mm.

#### Description.

*Head*. Head in dorsal view 2.0 times as wide as long; vertex abruptly sloping behind post ocelli; temple narrowed behind eyes; occipital carina complete, not crenulate; POL as long as OOL; toruli separated from each other; face setose along inner orbits; frons flat and smooth, with punctures above antennal sockets and setose; clypeus evenly convex; epistomal sulcus distinct; eye height 4.0 times length of malar space; malar sulcus absent. Antenna 1.1 times length of body; scape cylindrical, with apical rim simple; pedicel basally attenuate and apically broad; ratios of length to width  of antennal segments: 30 : 10; 15 : 8; 20 : 7; 25 : 7; 20 : 7; 20 : 7; 18 : 7; 18 : 9; 17 : 9; 17 : 9; 16 : 9; 16 : 9; 16 : 9; 15 : 9; 23 : 9.

*Mesosoma*. Mesosoma in dorsal view 0.8 times as long as width of head; pronotum angular in dorsal view, coarsely punctate anteriorly and setose; lateral side of pronotum with upper and anterior margins rugose-punctate, striate on lower 2/3 and smooth on upper 1/3; mesoscutum smooth and convex, with sparse long setae near humeral sulcus and notauli; notauli anteriorly present, oblique long and pit-like, weakly crenulate inside; humeral sulcus deep and crenulate, 1.5 times length of tegula; anterior scutellar pit transverse, crenulate inside, median longitudinal carina indistinct; scutellum smooth, posterior rim rounded; propodeum rugose, with transverse and longitudinal carinae distinct; mesopleuron smooth, with sparse setae on lower side; metapleuron rugose and setose.

*Wings*. Fore wing with costal, subcostal, basal, marginal, stigmal and postmarginal veins tubular; radial cell closed, 0.6 times length of marginal vein and 2.0 times as long as its height. Hind wing with a basal cell.

*Legs*. Fore and middle legs slender; hind tibia strongly incrassate.

*Metasoma*. Petiole transverse, with irregular longitudinal carinae; second tergite smooth and with a few scattered setae along lower side, median furrow short, extending to 0.2 length of second tergite; seventh tergite densely punctate; sutures between tergites complete and well impressed; sternites finely punctate and setose.

#### Colour.

Body black. Antenna entirely black. Legs brown, with coxae and trochanters black; fore and middle femora reddish brown with basal 0.3 and dorsal margin blackish; hind femur dark brown; hind tibia dark brown, with dorsal apical 3/4 brown. Wings hyaline, veins dark brown.

#### Male.

Unknown.

#### Distribution.

China (Yunnan).

#### Material examined.

Holotype, female. CHINA: Yunnan, Mt. Gaoligonshan (25.98°N, 98.80°E), 2006.VII.20-21, Zhong-shi Zhou, No. 200700989.

#### Diagnosis.

This species belongs to the *Ismarus halidayi*-group and is similar to *Ismarus longus* sp. n., but it can be easily distinguished from the latter by having the second flagellomere 3.5 times as long as wide (5.0 times in *Ismarus longus*); the radial cell of the fore wing 2.0 times as long as high (3.0 times); the seventh tergite densely punctate (finely mat); and the antenna uniformly black, all trochanters black (scape, pedicel and first flagellomere dark brown, trochanters brown).

#### Etymology.

The specific name refers to the black trochanters of this species.

**Figures 5–8.**
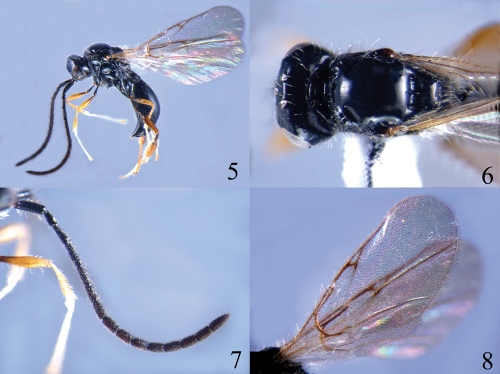
*Ismarus nigritrochanter* sp. n. **5** habitus, lateral view **6** head and thorax, dorsal view **7** antenna **8** fore wings.

### Ismarus parvicellus

Liu, Chen & Xu sp. n.

urn:lsid:zoobank.org:act:18E038AE-DB02-4891-95E0-5A7902BAFCE4

http://species-id.net/wiki/Ismarus_parvicellus

Figs 9–12 

#### Holotype.

Female. Body length 2.2 mm; fore wing length 1.8 mm.

*Head*. Head in dorsal view 2.0 times as wide as long; vertex abruptly sloping behind post ocelli; temple narrowed behind eyes; occipital carina strong and complete, not crenulate; POL as long as OOL; toruli separated from each other; face setose; clypeus evenly convex; epistomal sulcus distinct; eye height 7.0 times length of malar space; malar sulcus absent. Scape cylindrical, with apical rim simple; first to third flagellomeres attenuate basally, and gradually incrassate to apex; ratios of length to width of antennal segments: 20 : 5; 8 : 5; 11 : 4; 11 : 4; 9 : 4; 8 : 5; 8 : 5; 7 : 6; 7 : 6; 7 : 6; 6 : 6; 6 : 6; 6 : 6; 6 : 5; 10 : 5.

*Mesosoma*. Mesosoma in dorsal view 0.75 times as long as width of head; pronotum angular in dorsal view, anteriorly punctate and setose; lateral side of pronotum with anterior and upper margins coarsely punctate, lower half rugose punctate, posterior upper part smooth; mesoscutum smooth and convex; notauli present as small pits on anterior face; humeral sulcus strong, 1.4 times length of tegula; anterior scutellar pit transverse, crenulate inside, median longitudinal carina weak; scutellum smooth, posterior rim weakly concave and subtruncate; propodeum rugose and punctate, with transverse carinae and longitudinal carinae strong; mesopleuron mostly smooth and bare, with upper corner below tegula finely punctate and setose; metapleuron rugose and setose.

*Wings*. Fore wing with costal, subcostal, basal, marginal, stigmal and postmarginal veins tubular; distal part of median and cubital veins weakly pigmented; radial cell closed, 0.3 times length of marginal vein and 2.3 times as long as its height. Hind wing with a basal cell.

*Legs*. Fore and middle legs slender; hind tibia strongly incrassate.

*Metasoma*. Petiole transverse, rugose and with irregular longitudinal carinae; second tergite mostly smooth and scattered with a few setae along lower side, median furrow short, extending to 0.2 times length of second tergite; sutures between tergites complete and well impressed.

#### Colour.

Body black. Antenna yellowish brown but seventh to eighth flagellomeres blackish brown and ninth to thirteenth flagellomeres black. Legs brown. Wings hyaline, veins brown.

#### Male.

Unknown.

#### Distribution.

China (Hainan).

#### Material examined.

Holotype, female. CHINA: Hainan, Baisha County, Mt. Jiujialing (19.21°N, 109.45°E), 2010.VII.18, YPT, Hua-yan Chen, No. 20100013.

#### Diagnosis.

This species belongs to the *Ismarus halidayi*-group and can be distinguished from *Ismarus halidayi* by having the eye height 7.0 times length of the malar space (4.5 times in *Ismarus halidayi*); the radial cell of the fore wing small and 0.3 times length of marginal vein (radial cell as long as marginal vein); the distal part of the median and cubital veins weakly pigmented (median and cubital veins distinctly pigmented); the posterior rim of the scutellum weakly concave and subtruncate (posterior rim of scutellum round).

#### Etymology.

The specific name is derived from the Latin adjective ‘parvi’ (small) and ‘cell’, referring to the small radial cell of fore wing.

**Figures 9–12.**
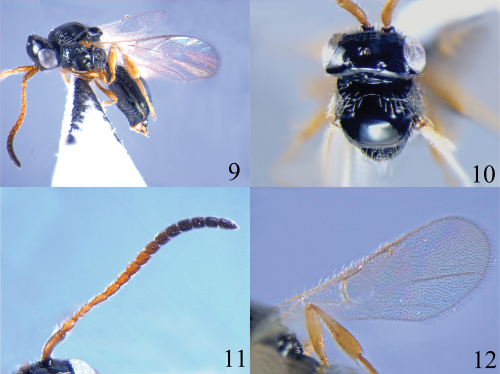
*Ismarus parvicellus* sp. n. **9** habitus, lateral view **10** head and thorax, dorsal view **11** antenna **12** fore wing.

### Ismarus dorsiger

(Haliday, 1831) New to China

http://species-id.net/wiki/Ismarus_dorsiger

Figs 13–16

Cinetus dorsiger Haliday, *in*Curtis 1831, British Entomology 3: 380
Betyla anomala Nees von Esenbeck 1834, Hymenopterorum ichneumonibus affinium monographiae, genera europaea et species illustrantes: 345. Synonymized by Foerster (1856).
Ismarus dorsiger :Haliday 1835, Entomological Magazine: 467. Generic transfer.
Ismarus neesii Foerster 1850, Verhandlungen des Naturhistorischen Vereins de Preussischen Rheinlande Westfalens: 286. Synonymized by Haliday (1857).
Ismarus dorsiger (Haliday, 1831): Johnson 1992, Memoirs of the American Entomological Institute 51: 260.

#### Material examined.

9 females, CHINA: Yunnan, Yongshan County, Huanghua Town (28.00ºN, 103.51ºE), 1500 m, 2010.X.8, Wei Dong.

#### Biology.

Hyperparasitoid of *Aphelopus* sp. of Dryinidae (Jervis, 1979).

#### Distribution.

China (Yunnan); England; Ireland; Spain; Russia.

#### Comments.

All specimens of this species were collected from a field with *Zanthoxylum bungeanum* plants (Sapindales: Rutaceae) in Northeast Yunnan together with some specimens of Dryinidae and Ceraphronidae.

**Figures 13–16.**
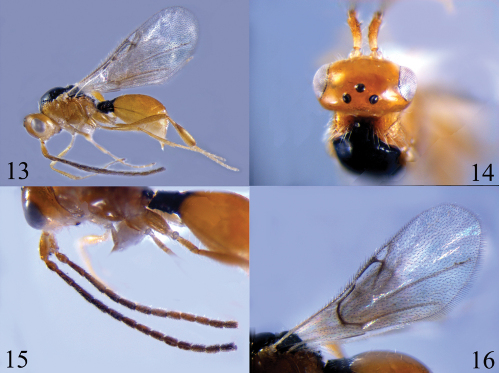
*Ismarus dorsiger* **13** habitus, lateral view **14** head, dorsal view **15** antennae **16** fore wing.

### Ismarus halidayi

Foerster, 1850 New to China

http://species-id.net/wiki/Ismarus_halidayi

Figs 17–20

Ismarus halidayi Foerster, 1850 Verhandlungen des Naturhistorischen Vereins de Preussischen Rheinlande Westfalens 7: 285.
Ismarus halidayi Foerster, 1850: Masner 1976, Canadian Entomologist 108: 1251.
Ismarus halidayi Foerster, 1850: Johnson 1992, Memoirs of the American Entomological Institute 51: 261.

#### Material examined.

2 females and 1 male, Ningxia, Mt. Liupanshan (35.40°N, 106.38°E), 2008.VII.11-12, Jie-min Yao, Nos. 200808622, 200808859, 200808017; 1 male, Ningxia, Mt. Liupanshan (35.40°N, 106.38°E), 2009.VII.3-14, Hua-yan Chen, No. 200903337; 1 female, Sichuan, Mt. Ermei (29.61°N, 103.36°E), 2009.VII.7, Jiang-li Tan, No. 200903977; 1 female, Sichuan, Luhuo (31.38°N, 100.66°E), 2009.VI.30, Jiang-li Tan, No. 200903953; 1 female, Guizhou, Mt. Fanjingshan (27.92°N, 108.70° E), 2100 m, 2001.VII.30, Yun Ma, No. 200109552; 1 female, Guizhou, Mt. Fanjingshan (27.92°N, 108.70° E), 1993.VII.12, Song-lin Yao, No. 936734; 1 female, Yunnan, Yongshan County, Huanghua Town, 1500 m (28.00°N, 103.51°E), 2010.X.8, Wei Dong; 1 male, Yunnan, Dali, Mt. Cangshan (25.63°N, 100.16°E), 2009.VI.5, Jiang-li Tan, No. 200901192; 1 male, Tibet, Milin (29.18°N, 94.20°E), 2009.VI.14, Jiang-li Tan, No.200902367.

#### Variation.

Some specimens from Yunnan (No. 200901192) and Tibet (No.200902367) with hind tarsus dark brown to black.

#### Distribution.

China (Ningxia, Sichuan, Guizhou, Yunnan, Tibet); Russia; England; Sweden; Finland.

**Figures 17–20.**
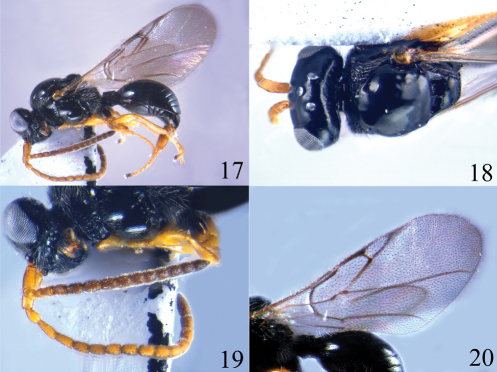
*Ismarus halidayi* **13** habitus, lateral view **14** head and thorax, dorsal view **15** antennae **16** fore wing.

## Key to species of genus *Ismarus* Haliday from China (based on females)

**Table:** 

1	Body pale yellowish; POL shorter than OOL	*Ismarus dorsiger* (Haliday, 1831)
–	Body entirely black; POL as long as OOL	2
2	Radial cell of fore wing as long as marginal vein; median furrow of second tergite long	*Ismarus halidayi* Foerster, 1850
–	Radial cell of fore wing distinctly shorter than marginal vein; median furrow of second tergite short	3
3	Second flagellomere 2.75 times as long as wide; apical flagellomere twice as long as wide; notauli present as small pits, smooth; radial cell of fore wing short, 0.3 times length of marginal vein; posterior rim of scutellum weakly concave and subtruncate	*Ismarus parvicellus* sp. n.
–	Second flagellomere 3.5–5.0 times as long as wide; apical flagellomere 2.5 times as long as wide; notauli present as oblique long pits and crenulate; radial cell of fore wing longer, 0.6 times length of marginal vein; posterior rim of scutellum round	4
4	Second flagellomere 3.5 times as long as wide; radial cell of fore wing 2.0 times as long as high; seventh tergite densely punctate; antenna uniformly black; all trochanters black	*Ismarus nigritrochanter* sp. n.
–	Second flagellomere 5.0 times as long as wide; radial cell of fore wing 3.0 times as long as high; seventh tergite finely mat; antenna black with scape brown, pedicel and first flagellomere dark brown; all trochanters brown	*Ismarus longus* sp. n.

## Supplementary Material

XML Treatment for Ismarus

XML Treatment for Ismarus longus

XML Treatment for Ismarus nigritrochanter

XML Treatment for Ismarus parvicellus

XML Treatment for Ismarus dorsiger

XML Treatment for Ismarus halidayi
